# Cytotoxic and apoptotic effects of etoposide and ellagic acid alone or with possible synergistic/additive combinations on a canine D-17 osteosarcoma cell line

**DOI:** 10.17221/24/2025-VETMED

**Published:** 2025-08-30

**Authors:** Gamze Sevri Ekren Asici, Funda Kiral, Aysegul Bildik, Pinar Alkim Ulutas

**Affiliations:** ^1^Department of Biochemistry, Faculty of Veterinary Medicine, Aydin Adnan Menderes University, Aydin, Turkiye

**Keywords:** acridine orange/ethidium bromide staining, Bax/Bcl-2 ratio, caspase 3, caspase 8, caspase 9

## Abstract

Osteosarcoma (OSA) is the most prevalent and aggressive malignancy of canine bones. Etoposide is an effective chemotherapeutic agent for cancer treatment, whereas ellagic acid is a naturally occurring compound with antioxidant and anticancer properties; however, both are inhibitors of the topoisomerase enzyme. In this study, the synergistic/additive effect of etoposide, known to have a growth-inhibitory impact in canine osteosarcoma (OSA) cells, and ellagic acid was investigated. The mechanism by which this effect inhibits cell growth at lower etoposide doses was also examined. The IC_50_ values of both agents were determined, and possible combination doses were generated accordingly and applied to canine OSA cells. The apoptotic effects of the combinations were evaluated based on DNA breaks and the activity levels of caspase 3, 8, and 9. These findings were supported by the expression levels of* Bcl-2, Bax,* and *Bid* genes*,* as well as the AO/EtBr staining method. The effects on cell cycle and proliferation were analysed through *survivin* and *NF-κβ* gene expressions. Antimetastatic effects were determined using invasion and migration assays. EA is a potential therapeutic agent for cancer treatment. In combination with ET, a higher anticancer efficacy was demonstrated compared to etoposide alone. Potential treatment side effects can be reduced by enabling the use of lower drug doses.

Osteosarcoma (OSA) originates from osteoblasts and cells that are responsible for forming the osteoid matrix, accounting for 80–85% of bone tumours (Wilson-Robles et al. 2019). OSA is a highly aggressive and invasive neoplasm, which, unfortunately, has a propensity to spread rapidly (Morello et al. 2011; Ludwig et al. 2024).

The current treatment options for canine OSA include limb amputation and limb-sparing tumour resection, followed by adjuvant chemotherapy. Platinum derivatives (cisplatin and carboplatin) and anthracycline antibiotics (doxorubicin) are commonly used in chemotherapy (Wright et al. 2019; Ludwig et al. 2024). OSA treatment is complex and often requires combination therapy (Li et al. 2021). The clinical results of these treatment methods (Morello et al. 2011) aimed at preventing metastasis and improving quality of life have failed to increase survival time (Wright et al. 2019). Although treatment options for cancer are growing daily, the survival rate of dogs afflicted with OSA remains low under the prevailing circumstances (Ong et al. 2017a). Despite the administration of chemotherapy, metastasis continues to develop due to the poor prognosis, with distant metastasis being the most common cause of death (Ong et al. 2017a; Ludwig et al. 2024).

In addition to metastasis, tumour resistance to chemotherapeutic drugs is another challenge. The development of resistance to doxorubicin-based chemotherapy limits the curative effects of chemotherapy in OSA. Different treatment protocols need to be investigated due to both the inability to prevent metastasis and the development of drug resistance (Li et al. 2021), and the enhancement of survival rates (Wilson-Robles et al. 2019).

Etoposide (ET) is a potent anti-mitotic and anti-neoplastic agent that has been used clinically for over two decades. The primary mechanism of action of ET is the inhibition of topoisomerase II (Kim et al. 2022), thereby inhibiting DNA replication and transcription by causing single-and/or double-stranded breaks to accumulate in the double strand. Accumulation of breaks stimulates apoptosis. Moreover, ET mainly exerts its effects during the G2 and S phases of the cell cycle (Bender et al. 2008). ET has been used in some human OSA treatment protocols with varying results (Le Deley et al. 2007; O’Kane et al. 2015; Schwartz et al. 2016). Although pharmacokinetic and toxicological studies of ET in dogs are available (Hohenhaus and Matus 1990), studies on relapsed canine lymphoma and haemangiosarcoma, alone or in combination with other agents, have shown that ET may also have anticancer activity in dogs (Hohenhaus and Matus 1990; Boye et al. 2017). However, studies on its anticancer efficacy, alone or in combination with OSA, are limited (Boye et al. 2017; Ong et al. 2017a; Ong et al. 2017b; Ong et al. 2019).

Ellagic acid (EA) is a naturally occurring phenolic lactone compound found in high concentrations in many fruits, such as strawberries, raspberries, cranberries, grapes, and green tea (Chen et al. 2001; Han et al. 2006). Many studies have proven that EA has antiproliferative, anticancer, and antiangiogenesis effects on cancer development and invasion pathways by modulating signalling pathways (Wnt/β-catenin; PI3K/Akt) and molecular targets controlling the cell cycle (including p53, pRB, p16 and p21, cyclins, cyclin-dependent kinases) (Li et al. 2005; Fang et al. 2015; Yousef et al. 2016; Wang et al. 2017; Gordon et al. 2018). This effect has been demonstrated *in vitro* in human ovarian (Chung et al. 2013), pancreatic (Zhao et al. 2013), colon (Zhao et al. 2020), lung (Liu et al. 2018) cancer, and osteosarcoma (Xu et al. 2018) cell lines.

In recent years, the tendency towards combined treatments has increased due to the failure to observe the targeted effects on antiproliferative, anticancer, and anti-metastatic mechanisms during treatment with chemotherapy drugs administered alone. Particularly in domestic animals, treatments that block tumour cell proliferation, induce apoptosis, block DNA damage caused by oxidative stress and carcinogens, interfere with inflammation, inhibit angiogenesis, and prevent tumour progression and metastasis, either alone or in combination, are limited. A substantial challenge in the therapy of canine OSA is its high metastatic potential. Current combination therapies focus on antiapoptotic and antiproliferative effects. However, in the current study, targeted combination therapy approaches were evaluated not only for these effects but also for their antimetastatic potential.

Therefore, new therapeutic strategies are required to manage and treat canine OSA (Poon et al. 2020). A limited number of studies have suggested that combining an antioxidant with a chemotherapeutic agent may be a potential therapeutic approach for OSA. In the current study, the possible effects of ellagic acid supplementation on etoposide chemotherapy were investigated to induce apoptosis induced by high-dose chemotherapy and reduce the side effects that may develop due to these doses. The potential additive or synergistic antineoplastic, anti-invasive, and apoptotic effects of a combination of ellagic acid and etoposide, both topoisomerase II inhibitors, were evaluated in canine OSA cell lines. The identification of possible synergistic interactions between ellagic acid and etoposide may improve the therapeutic efficacy of OSA treatment.

## MATERIAL AND METHODS

### Cell culture

The canine osteosarcoma cell line, D-17 (CCL-183), was purchased from American Type Culture Collection (ATCC; Manassas, VA, USA). Canine OSA D-17 (CCL-183) cells were cultured in Eagles minimal essential medium (EMEM, Sigma M4655; (St. Louis, MO, USA) supplemented with 10% foetal bovine serum (FBS, Sigma F0804, non-USA origin), 1% non-essential amino acids (Sigma M7145), 0.11 g/l sodium pyruvate, and 0.1% Penicillin/Streptomycin at 10 000 U/ml (Gibco). The cells were grown at 37 °C in a humidified atmosphere containing 5% CO_2_. The number of cells used in the experiments varied between 4 and 8.

### Effect of ellagic acid (EA), Etoposide (ET), and their combination on cell viability

D-17 canine OSA cells were seeded into 96-well cell culture plates (5 × 10^3^ cells) and incubated at 37 °C in 5% CO_2_ for all cells to adhere and reach confluence (20–24 h). The same procedure was applied to all cytotoxic assays for all agents and combinations used in the study. Each compound and its combinations were tested in four biological replicates and three independent replicates. The viability of the 0 μM EA/ET (control) sample was accepted as 100% for all periods.

A stock solution of ellagic acid (Sigma E2250, USA) was prepared in cell medium containing DMSO. The stock EA solution was diluted with EMEM supplemented with concentrations of 5 μM, 12.5 μM, 25 μM, 50 μM, 100 μM, 150 μM, and 200 μM such that the DMSO concentration did not exceed 1%. The concentrations of 1.25 μM, 2.5 μM, 3.75 μM, 5 μM, 7.5 μM, 10 μM, 15 μM, 20 μM, 25 μM, 50 μM, 75 μM, and 100 μM were prepared from an Etoposide (Actavis, UK) stock solution using cell media. D-17 canine OSA cells were incubated in these concentrations for 24, 48, and 72 h at 37 °C in 95% humidified air and 5% CO_2_. At the end of the incubation period, cytotoxicity analysis was performed using WST-1 (Roche-11644807001). The data obtained from the WST-1 cytotoxicity test were used to calculate IC_50_ values in GraphPad Prism v5.0 (GraphPad Software; La Jolla, CA, USA).

### Cell invasion and migration

The invasion and migration capacity of D-17 canine OSA cells treated with ET, EA, and their combinations were determined using a 24-well invasion chamber (Falcon HTS 24-Multiwell Insert Systems, Corning^®^, USA). The 8-μm pore diameter of the invasion chamber was compatible with the cell types used in the experiments. For the invasion analysis, the insert systems were covered with Corning Matrigel Basement Membrane Matrix (354234, USA), which functions as a basement membrane *in vitro* and prevents cell invasion by clogging the pores of the membrane.

After optimisation studies, the cells were incubated for 48 h in the determined combinations for invasion analysis, starved for 16 h, and inserted into the insert system to show their effects on invasion. Each application of ET, EA, and the combination was performed in triplicate. The cells that invaded the membrane were fixed in formaldehyde. After fixation, the sections were washed with phosphate-buffered saline (PBS) and kept in crystal violet staining solution (prepared as 0.5 g in ethanol) for 15 minutes. The inserts were then dried. At least three different areas of each membrane were imaged and photographed using a camera-integrated light microscope (Olympus). Invading cells were counted using ImageJ software (v1.49; National Institutes of Health, USA). Graphs were prepared to evaluate mean cell numbers and standard deviations.

### Demonstration of DNA fragmentation using enzyme-linked immunosorbent assay (ELISA)

The Cell Death Detection ELISA Plus Kit (Roche, Germany) was used to determine the apoptotic effects of EA and ET alone and in combination, according to the manufacturer’s protocol. Canine OSA cells were seeded at 5 × 10^5^ cells per well in six-well cell plates in triplicate and incubated at 37 °C for 24 h to allow cells to adhere. Etoposide (7.5, 10, 15, 20 μM) and/or EA at 25 and 50 μM were added to the wells, and the cells were incubated for 24 and 48 hours. After incubation, 1 × 10^4^ cells were counted, and lysis buffer was added to obtain the nuclear extract. Apoptotic DNA fragments from each sample were analysed using ELISA. Enrichment factors for the other samples were calculated proportionally, with mono- and oligonucleosome enrichment indicating DNA breaks indicated in the kit, assuming 1.00 for the sample without agent treatment (control cells).

### Evaluation of apoptosis by determining Caspase 3, 8 and 9 using ELISA

Cells (5 × 10^4^ cells/well) were seeded in six-well plates. Cells were treated with ET, EA, and their combinations when adherent growth was observed, and then incubated for 24 and 48 hours. Floating and trypsinised adherent cells were collected and lysed with RIPA buffer. Analyses were performed immediately. A canine-specific ELISA kit (FineTest, Canine Caspase 3, Caspase 8, Caspase 9; P.R. China) suitable for cell culture was used to determine caspase 3, -8, and -9 levels in cell lysates. Two independent experiments were performed in triplicate.

### Ratios of viable, apoptotic, and necrotic cells

Approximately 5 × 10^5^ canine OSA cells per well were seeded in six-well plates and allowed to adhere. Then the cells were treated with ET (7.5, 10, 15, 20 μM) and/or EA at concentrations of 25 and 50 μM for 24 and 48 hours. D-17 canine OSA cells were examined under a fluorescence-attached microscope (Olympus BX53, Japan) by staining with acridine orange (Biotium, USA) (100 μg/ml) and ethidium bromide (Biotium, USA) (100 μg/ml) (Kasibhatla et al. 2006).

### Determination of apoptotic gene expression using reverse transcription polymerase chain reaction (RT-PCR)

#### PRIMERS

Primers for canine-specific *Bcl-2*, *Bax*, *survivin*, *Bid*, *NF-κβ*, and housekeeping gene glyceraldehyde 3-phosphate dehydrogenase (*dogGAPDH*) were used for expression analysis using RT-PCR (Table 1). The primers were designed using Primer3 v.0.4.0. and the *Canis lupus familiaris* gene sequences deposited in public databases. Primer dimer formation, non-specific amplification, and self-priming were checked using IDT Oligo Analyser software.

**Table 1:** Primer sequences used for qRT-PCR

RefSeq number	Gene	Forward primer (5'→3')	Revers primer (5'→3')
NM_001003142.2	*GAPDH*	AGTCAAGGCTGAGAACGGGAAA	TCCACAACATACTCAGCACCAGC
NM_001002949.1	*Bcl-2*	CATGCCAAGAGGGAAACACCAGAA	GTGCTTTGCATTCTTGGATGAGGG
NM_001003011.1	*Bax*	TTCCGAGTGGCAGCTGAGATGTTT	TGCTGGCAAAGTAGAAGAGGGCAA
NM_001251938.1	*Bid*	AATTTGCTAGTGTTTGGCTTCCTC	ATCGTCGTAGTCCTCCTTCAG
NM_001003348.1	*survivin*	CCCAGTGTTTCTTCTGCTTCAA	AGAAAGGAAAGCACAACCAGATG
XM_022405652.1	*NF-κB*	ATCCCATCTTTGACAACCGT	CACTTCAATGTCCTCTTTCTG

#### RNA ISOLATION AND DETERMINATION OF GENE EXPRESSION LEVEL

RNA was isolated using a commercial RNA isolation kit (RiboEX, GeneAll, Republic of Korea). RNA samples were converted to cDNA using a cDNA synthesis kit (Transcriptor High Fidelity cDNA Synthesis Kit, Roche, Germany) according to the manufacturer’s instructions. Expression levels of genes were determined using real-time quantitative reverse transcription PCR (qRT-PCR) (GeneAll, Korea) with a LightCycler 480 device (Roche, Germany).

Cycling conditions were set as an initial denaturation step for 10 min at 95 °C, followed by 40 cycles of 20 s at 95 °C for template denaturation, 20 s for the annealing phase at 60 °C for *Bcl-2* and *Bax* genes, at 55 °C for *survivin*, *Bid,* and *NF-κβ* genes, and 15 s at 72 °C for the extension. Each cDNA sample was tested three times with three biological replicates, and the cycle threshold (C_t_) was determined. The expression of target genes was normalised to that of *GAPDH*. Following the normalisation process, the ΔΔCt method developed by Livak and Schmittgen (2001) was used to calculate the differences between groups.

### Statistical analysis

Data were collected and evaluated using SPSS for Windows v22 (SPSS? USA). The Shapiro–Wilk test was used to examine the conformity of the variables to a normal distribution. Descriptive analyses used mean and standard deviation values for both normally and non-normally distributed variables.

The conformity of the variables to a normal distribution, based on both general and categorical data, was visually evaluated using histograms and probability graphs, as well as analytically using Kolmogorov–Smirnov and Shapiro–Wilk tests. Descriptive statistics were presented as mean and standard error values. The null hypothesis (*H*_0_) of the study was that there is no significant difference in the proliferation, apoptosis, and metastasis parameters of etoposide and ellagic acid combinations at different doses in the canine osteosarcoma cell line. The alternative hypothesis (*H*_1_) predicted a significant difference in the apoptotic and metastatic parameters of these combinations at various doses. A one-way analysis of variance (ANOVA) was used to evaluate whether there was a statistically significant difference in the means of the groups receiving ellagic acid, etoposide, or their combination. The homogeneity of variances was tested using Levene’s test. In cases where the ANOVA indicated significant differences among groups, a post-hoc Tukey’s test was applied. Statistical significance was set at *P* < 0.05.

Because the data obtained from the WST-1 cell proliferation experiment did not conform to a normal distribution, the combination analysis data were log-transformed to ensure homogeneity of variance (especially in ANOVA), and reduce the effect of outliers, and a One-Way ANOVA was conducted.

## RESULTS

### Effects of EA and ET on cell viability

**Figure 1:**
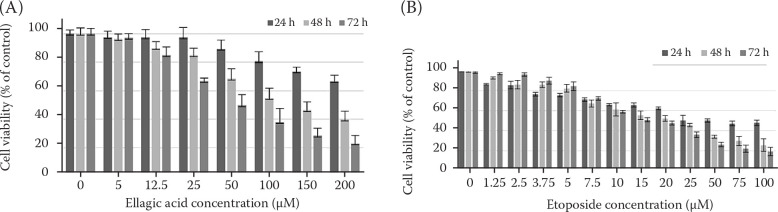
Time-dependent effect of different doses of EA (A) and ET (B) on D-17 canine OSA cells EA = ellagic acid; ET = etoposide; OSA = osteosarcoma

A viability graph was generated using the WST-1 assay results by applying 5–200 μM concentrations of ellagic acid (Figure 1A) and 1.25–100 μM concentrations of ET (Figure 1B) to D-17 canine OSA cells incubated for 24, 48, and 72 h.

Whereas EA demonstrated no antiproliferative effect at low doses (5, 12.5 μM), a decline in cell viability was induced at higher doses. At the highest dose (200 μM) for 24 h, there was 35% inhibition of cell viability. Therefore, IC_50_ values could not be calculated. The IC_50_ value of EA was determined to be 30.72 ± 1.45 μM at 48 h and 16.91 ± 0.89 μM at 72 hours. After 48 h, >50 μM responded to the control group. At 72 h, all concentrations applied except 5 μM differed from the control.

The IC_50_ value of ET concentrations was 19.3 ± 0.97 μM at 24 h, 13 ± 0.43 μM at 48 h, and 11.36 ± 0.61 μM at 72 hours. At higher concentrations (7.5, 10, 15, 20 μM), a decrease in cell viability was found compared to the control, depending on the incubation time (*P* < 0.05).

### ET and EA on WST-1 cells

ET concentrations of 7.5, 10, 15, and 20 μM were used. Greater than 25 μM resulted in cell viability inhibition of >50%, compared to the control. The maximum plasma concentrations after intravenous administration of 50 mg/m^2^ etoposide were 1.2–16.4 μM in dogs with cancer (Flory et al. 2008). Concentrations close to and just above the IC_50_ dose were determined for 24 h, and the interactions of possible combinations were determined with the data obtained from the WST-1 analysis using CompuSyn v1.0 (ComboSyn Inc., Paramus, NJ, USA). The combination indices (CI) results were evaluated as follows: CI = 1 indicated an additive effect, CI < 1 indicated a synergistic effect, and CI > 1 indicated antagonism (Chou 2010). The CI values of the combination of ET and EA after 24, 48, and 72 h of incubation were less than 1 (CI < 1) (Table 2).

**Table 2:** Effect of ellagic acid and etoposide combinations on dose-dependent cell viability in the D-17 canine osteosarcoma cell line at 24, 48 and 72 h

Combinations	24-h			48-h			72-h	
	% vitality	CI values		% vitality	CI values		% vitality	CI values
Control	100 ± 2.15	–		100 ± 3.12	–		100 ± 3.47	–
25 EA	79.73 ± 2.48	–		59.35 ± 2.47	–		52.41 ± 2.14	–
50 EA	69.54 ± 2.78	–		43.66 ± 3.98	–		25.83 ± 2.34	–
7.5 ET	79.02 ± 3.01	–		59.00 ± 4.03	–		35.89 ± 4.12	–
7.5 ET × 25 EA	70.90 ± 1.75	0.914		46.09 ± 3.46	0.821		26.11 ± 1.03	0.667
7.5 ET × 50 EA	54.24 ± 2.11	0.455		31.86 ± 2.14	0.900		15.74 ± 1.78	0.647
10 ET	75.47 ± 1.52	–		51.56 ± 2.97	–		32.36 ± 2.35	–
10 ET × 25 EA	65.25 ± 2.36	0.809		46.96 ± 2.47	0.868		24.85 ± 2.47	0.541
10 ET × 50 EA	54.15 ± 4.23	0.509		31.80 ±2.63	0.924		15.44 ± 2.46	0.539
15 ET	70.03 ± 2.63	–		47.51 ± 2.15	–		31.89 ± 3.45	–
15 ET × 25 EA	65.58 ± 3.12	0.904		42.61 ± 3.82	0.717		21.50 ± 2.14	0.704
15 ET × 50 EA	54.91 ± 2.46	0.658		31.83 ± 3.47	0.755		16.18 ± 3.25	0.697
20 ET	65.92 ± 3.26	–		45.73 ± 4.02	–		29.79 ± 2.78	–
20 ET × 25 EA	64.57 ± 2.96	1.089		40.40 ± 2.58	0.790		20.71 ± 2.46	0.731
20 ET × 50 EA	56.45 ± 3.15	0.665		30.41 ± 2.14	0.606		16.08 ± 1.65	0.734

CI = combination indices; EA = ellagic acid; ET = etoposide

### Cell invasion and migration

When invasion and migration studies were performed on cells exposed for 24 h, the cell numbers were too low for counting. Based on the WST-1 analyses, the combinations substantially reduced the 72-h viability rates and affected the reproducibility of the analysis. Therefore, a 48-h incubation was found to be appropriate.

**Figure 2:**
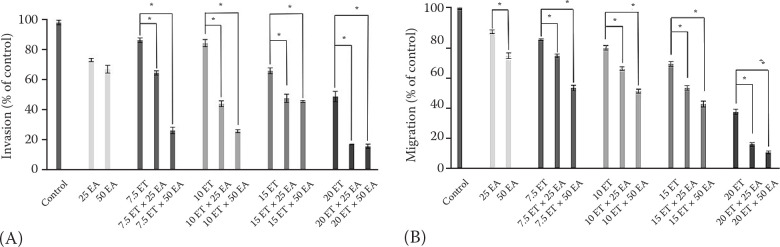
Effect of EA, ET and combination doses on the invasion (A) and migration (B) ability of cells in the D-17 canine OSA cell line The asterisks indicate significant differences between the groups analysed by the one-way ANOVA with Tukey’s test (*P < 0.001) EA = ellagic acid; ET = etoposide; OSA = osteosarcoma

Etoposide caused a statistically significant decrease in the ability to invade cells in a dose- and time-dependent manner. Compared to the control group, the invasion capacities of cells treated with 25 μM and 50 μM EA were 74.5% and 68%, respectively. In combination studies in which ET was applied together with EA, EA increased the suppressive effect of ET on the invasion ability of cells (except for 25EA and 7.5 ET + 25 EA) (*P* < 0.001; Figure 2A).

There was a decrease in the migration of D-17 canine OSA cells after treatment with increasing doses and durations of ET (*P* < 0.001; Figure 2B). Compared to the control group, the migration capacities of cells treated with 25 μM and 50 μM EA were 85% and 70%, respectively (*P* < 0.001). The migration ability of cells treated with combined doses of EA and ET was suppressed compared to that of cells treated with EA and ET alone (*P* < 0.001).

### Analysis of DNA fragmentation

**Figure 3:**
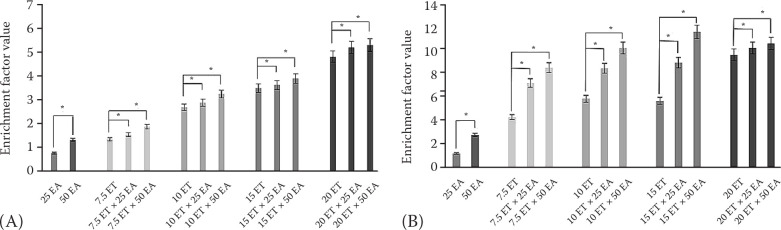
Apoptotic DNA breaks in D-17 canine OSA cells with EA, ET and EA + ET combinations after 24 h (A) and 48 h (B) incubation The asterisks indicate significant differences between the groups analysed by the one-way ANOVA with Tukey’s test (*P < 0.001) EA = ellagic acid; ET = etoposide; OSA = osteosarcoma

An increase in DNA breaks was observed with increasing ET dosage at the end of 24- and 48-h of incubation compared to the control (*P* < 0.001; Figure 3A,B). A similar increase in DNA breaks was observed with increasing incubation time at 25 μM and 50 μM ET (*P* < 0.001; Figure 3A,B). When the effects of ET in combination with EA and those of ET and EA alone were compared, all combinations increased DNA breaks and consequently showed a synergistic effect in inducing apoptosis.

### Ratios of apoptotic/necrotic/viable cells

**Figure 4:**
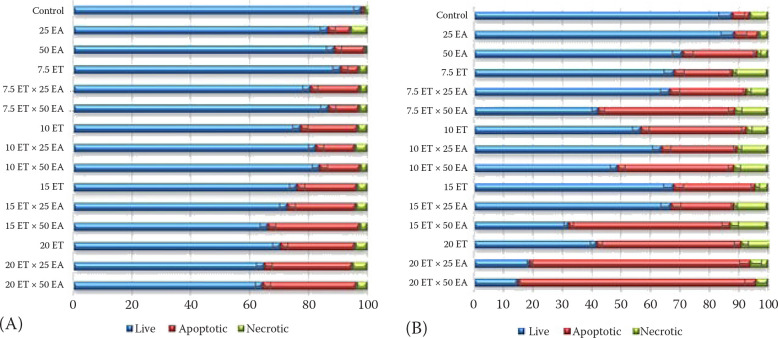
Live/apoptotic/necrotic D-17 cell ratios determined as a result of 24 h (A) and 48 h (B) incubation of canine OSA cells with EA, ET and EA + ET combinations EA = ellagic acid; ET = etoposide; OSA = osteosarcoma

There were no changes in necrotic cell numbers throughout the first 24 h with increasing doses of ET. The number of apoptotic cells increased substantially with the ET concentration. A similar result was observed after the application of EA. When the applied combinations were evaluated based on ET concentrations alone, they showed a synergistic effect except for the combination of 10 μM ET (Figure 4A). In 10 μM and 15 μM ET treatment, fewer necrotic cells were formed compared to other ET concentrations, whereas more cells approached apoptosis. Following EA treatment, the number of necrotic cells did not change in a dose-dependent manner, whereas the number of apoptotic cells increased. ET in combination with EA showed a dose-dependent synergistic effect, except for combinations of 10 μM and 15 μM ET with 25 μM EA (Figure 4B).

### Assessment of apoptosis using caspase 3, 8 and 9 in ELISA

Changes in caspase 3, 8, and 9 levels resulting from the incubation of D-17 OSA cells with EA, ET, and EA + ET combination for 24 and 48 h were determined by calculating them according to the standard graph in percent ng/ml.

**Figure 5:**
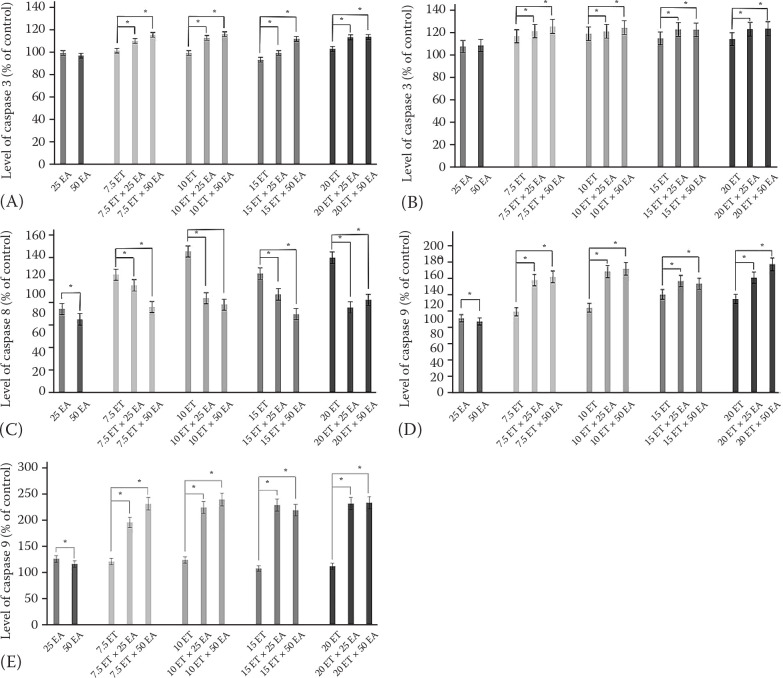
Dose-dependent change in caspase 3 level after 24 h (A) and 48 h (B) of incubation (*P < 0.001). Dose-dependent change in caspase 8 level after 48 h of incubation (*P < 0.001). (C) Dose-dependent change in caspase 9 level after 24 h (D) and 48 h (E) of incubation The asterisks indicate significant differences between the groups analysed by the one-way ANOVA with Tukey’s test (*P < 0.001) EA = ellagic acid; ET = etoposide

At the end of 24 h of incubation, an increase in caspase 3 levels was observed in the cells to which EA and ET in combination were applied compared to the cells treated individually (*P* < 0.001; Figure 5A). These increased levels were more evident at the end of the 48-h incubation (*P* < 0.001; Figure 5B). At the end of the 24-h incubation, caspase 8 levels fluctuated in the cells to which EA and ET combination doses were applied, compared to the cells to which ET was given alone. After 48 h, caspase 8 decreased in the combination doses compared to ET alone (*P* < 0.001; Figure 5C).

After 24 h of incubation, caspase 9 levels increased with EA and ET in combination (*P* < 0.001; Figure 5D). These increases were more pronounced after 48 h (*P* < 0.001; Figure 5E).

### Apoptosis as indicated by *Bax*/*Bcl-2* using qRT-PCR

**Figure 6:**
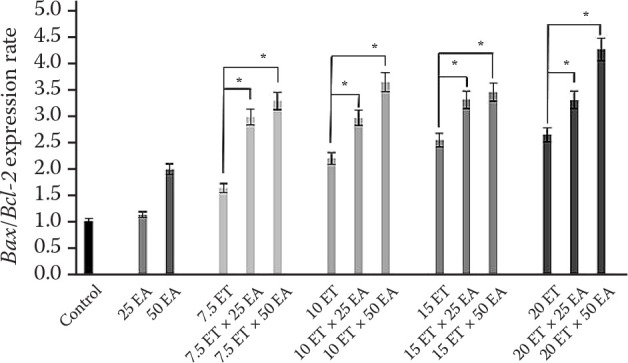
Dose-dependent change in *Bax*/*Bcl-2* ratio after 48 h of incubation The asterisks indicate significant differences between the groups analysed by the one-way ANOVA with Tukey’s test (*P < 0.001) EA = ellagic acid; ET = etoposide

After 24 h of incubation, an increase in the *Bax*/*Bcl-2* ratio was observed in cells treated with the combination dose compared to cells treated with EA and ET individually (Figure 6). However, this increase was significant only between 15 μM and 20 μM ET and combination doses (*P* < 0.05). After a 48-h incubation, *Bax*/*Bcl-2* increased at all combination doses compared to EA and ET individually (*P* < 0.001). In cells treated with 25 μM EA, a 1.05-fold increase in the *Bax*/*Bcl-2* ratio was obtained after 24 h, and a 1.13-fold increase was obtained after 48 hours. In cells treated with 50 μM EA, a 1.56-fold increase in the *Bax*/*Bcl-2* ratio was determined after 24 h, and this doubled after 48 hours. *Bax*/*Bcl-2* was similar after 24 h, but different after 48 h (*P* < 0.001).

### mRNA expression levels in D-17 canine OSA cells

**Figure 7:**
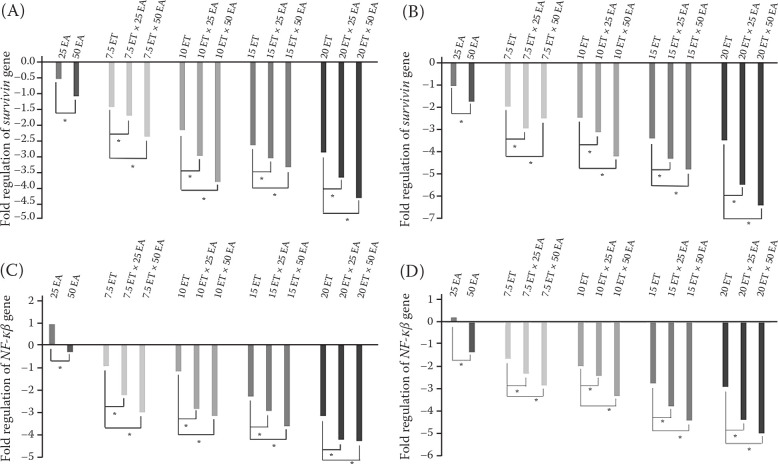
Change in the expression of the *survivin* gene after 24 hours (A) and 48 hours (B) of incubation. Change in the expression of the *NF-κβ* gene after 24 h (C) and 48 h (D) of incubation The asterisks indicate significant differences between the groups analysed by the one-way ANOVA with Tukey’s test (*P < 0.001) EA = ellagic acid; ET = etoposide

A quantitative decrease was observed in *survivin* in the D-17 canine OSA cell line compared to the control at all doses applied until 24 h (*P* < 0.001; Figure 7A). This decrease was more evident at 48 h (*P < *0.001; Figure 7B).

At the end of the 24- and 48-h incubation, a decrease in the expression of *NF-κβ* gene over the control was observed with EA and ET, and combination doses, depending on the dose and time (*P* < 0.001; Figure 7C,D). Although there was no decrease in the 25 μM EA alone in the first 24 h, a 0.8-fold decrease compared to the control was observed after 48 hours. For 50 μM EA alone, a reduction of 0.3-fold and 1.33-fold was determined after 24 and 48 h, respectively.

According to the RT-PCR results of D-17 OSA cells after 24 and 48 h of incubation with EA, ET, and the EA + ET combination, no statistically significant difference was found in the dose-dependent change in *Bid* expression (data not shown).

## DISCUSSION

Several cancer studies, including those on canine lymphoma and hemangiosarcoma, have demonstrated the effectiveness of ET as a single agent or in combination with other antineoplastic drugs. ET was selected for the *in* *vitro* study because use of this chemotherapeutic agent in veterinary oncology is currently limited (Hohenhaus and Matus 1990; Lana et al. 2007).

Ellagic acid applied to normal cells at concentrations lower than its cytotoxic effect (IC_50_) has no effect on cell proliferation, has an antioxidant effect, neutralises the reactive metabolites of free oxygen radicals and carcinogens, protects against apoptosis, and therefore prevents the onset of cancer (Zhang et al. 2014). EA reduces proliferation and induces apoptosis in breast, colon, and bladder cancer (Cizmarikova et al. 2023), human osteogenic sarcoma (HOS) (Han et al. 2006). To date, no studies have examined the antiproliferative effects of EA on canine OSA cell lines.

Our study aimed to determine whether the combination of ET with EA, a topoisomerase enzyme inhibitor, enhances the chemotherapeutic and antimetastatic efficacy of ET, which has recently been investigated for its potential use in canine OSA, in addition to cisplatin, carboplatin, and doxorubicin.

The effect of ET on cell viability has been investigated in numerous cell lines, yielding different IC50 values. The IC_50_ values of MG-63 and Saos-2 human OSA cell lines exposed to ET after 48 h of incubation were 21.5 and 9.6 μM, respectively (Ferreira de Oliveira et al. 2018). Coutinho et al. (2017) in their studies with human OSA cell line, U2OS, reported IC_50_ values as 30 μM (critical violet staining) and 24 μM (MTT results). They calculated IC_50_ values as 0.18 ± 0.02 μM, 0.18 ± 0.01 μM, and 0.32 ± 0.06 μM after 72 h of ET treatment to HMPOS, POS, and HOS in canine OSA cell lines, respectively (Ong et al. 2016). Poradowski and Obminska-Mrukowicz (2019) applied ET to D-17 canine OSA cells and human U2OS cells for 72 h and found IC_50_ values of 6.27 ± 0.31 μg/ml and 2.72 ± 0.51 μg/ml, respectively. We exposed D-17 canine OSA cells to varying doses of ET and calculated IC_50_ values as 19.3 ± 0.97 μM, 13 ± 0.43, 11.36 ± 0.61 μM after 24, 48 and 72 h of incubation, respectively.

The potential cytotoxic and anti-proliferative activities of EA have been evaluated in human MCF-7 breast, Hs 578T breast, Caco-2 colon, and DU 145 prostate cancer cells (Losso et al. 2004). In a study with Saos-2 and MG63 human OSA cells, as well as other cancers, the effective concentration was found to be 20 μM (Xu et al. 2018). In osteogenic sarcoma cells, the effective concentrations were 20 and 100 μg/ml (Han et al. 2006). However, its antiproliferative effects in canine OSA cell lines have not been investigated. However, when studies on human OSA cell lines were examined, the responses were similar to our data.

Several studies have demonstrated that ET inhibits cell migration and invasion (Ghavami et al. 2023; Yu et al. 2023). Xu et al. (2018) reported that EA inhibits the metastasis and invasion of Saos-2 and MG63 cells in their study. In the current study, EA reduced the invasiveness and migration ability of D-17 canine OSA lines when applied alone or in combination with ET at specified doses.

Apoptosis is activated via two mechanisms. These are the extrinsic pathways activated by the binding of death ligands to death receptors and the intrinsic pathways activated by intracellular signalling. Both these pathways activate a series of caspases to target and degrade key cellular proteins (Bildik and Bayar 2018). The extrinsic apoptotic pathway is activated by the binding of death receptors, which are transmembrane proteins and members of the tumour necrosis factor receptor (TNFR) superfamily, on the cell surface by ligands belonging to the tumour necrosis factor (TNF) superfamily (Jan and Chaudhry 2019). The intrinsic pathway refers to the mitochondrial-mediated apoptotic pathway, which is mediated by Bax/Bak insertion into the mitochondrial membrane and the subsequent release of cytochrome c from the mitochondrial intermembrane space into the cytosol. Cytochrome c binds to Apaf-1 and procaspase 9 to form apoptosomes. The apoptosome triggers caspase 9, followed by activation of the caspase 3 signalling cascade, leading to cell destruction and ultimately apoptosis (Jin and El-Deiry 2005; Bildik and Bayar 2018). Apoptosis is a tightly regulated process involving changes in the expression of individual genes. Because the expression level of the active form of caspase 3 is commonly used to determine apoptosis (Forest et al. 2005), the amount of caspase 3 can provide information about the level of apoptosis.

Studies have been conducted to elucidate the molecular mechanisms underlying the apoptotic effects of ET in various cancer cells (Jamil et al. 2015). Wang et al. (2006) demonstrated that high ET concentrations may trigger caspase-mediated apoptosis via the cytochrome c/caspase 9 pathway. ET increased caspase 3 and 9 activity in leukaemia cell lines but did not affect caspase 8 (Mahbub et al. 2015).

The Fas ligand (FasL) pathway is also involved in ET-induced apoptosis (Kaufmann and Earnshaw 2000). Caspase 8 may be activated and interact with effector caspases such as caspase 3 (Montecucco et al. 2015). Similarly, Lin et al. (2004) and Day et al. (2009) reported increased caspase 8 levels in a neuroblastoma cell line following etoposide treatment. In the current study, although there was no statistical difference in the first 24 h of ET administration over the control, there was a substantial increase in caspase 3 level at the applied doses compared to the control at 48 hours.

Thus, it can be concluded that high doses of ET increase caspase 3 levels in canine OSA cells. There was an increase in caspase 9 levels depending on the ET concentration applied in the first 24 hours. In 7,5 and 10 μM ET applications, caspase 9 continued to increase at 48 h, whereas at 15 μM ET, the level decreased compared to 24 h, and at 20 μM ET, caspase 9 was similar.

A statistically non-significant increase in caspase 8 was detected at all ET concentrations, except 20 μM ET, in the first 24 hours. At 48 h, there was a statistically significant increase in all treatments. In summary, ET application induced apoptosis in canine OSA cells by increasing the activity of effector caspase 3 through the activation of initiator caspases 8 and 9.

EA induces apoptosis by activating caspase 3 (Mertens-Talcott et al. 2006; Day et al. 2009). Alfredsson et al. (2014) demonstrated that EA induced caspase 9 and caspase 3 activation in human neuroblastoma cells, resulting from increased mitochondrial membrane permeability in a dose- and time-dependent manner. Similarly, Larrosa et al. (2006) reported that Caco-2 cells induced caspase 9 as the initiator caspase and caspase 3 as the effector caspase. Li et al. (2005) reported that EA application to a bladder cancer cell line did not affect the level of caspase 8 and that caspase 8-independent apoptosis occurred.

In the current study, the increase due to ET was ineffective in the applied combinations, and EA may have caused a decrease in caspase 8 levels by inducing apoptosis independent of caspase 8. The identified combinations had an antagonistic effect on caspase 8 activity. When *Bid* gene expression levels were examined, the absence of a significant change in cells treated with EA and ET supported these results.

It can be concluded that the combination of EA and ET had a synergistic effect compared to individual treatment, with caspase 9 as the initiator caspase and caspase 3 as the effector caspase; the combination induced more apoptosis.

The Bcl-2 family consists of anti-apoptotic members such as Bcl-2 and pro-apoptotic members such as Bax, which are the most important regulators of apoptosis (Deveraux and Reed 1999). The Bcl-2/Bax signalling pathway is involved in the regulation of apoptosis (Schwartz et al. 2016). In our study, the expression level of *Bax* increased in a time-dependent manner with increasing ET concentration in D-17 canine OSA cells, whereas Bcl-2 levels generally showed no statistically significant change. EA increased the expression level of *Bax* and decreased that of *BCL-2* compared to the control group. Similarly, studies on human ovarian cancer (Chung et al. 2013), prostate cancer (Naiki-Ito et al. 2015), and OSA cells (Han et al. 2006) have reported that EA can downregulate *Bcl-2* expression, upregulate *Bax* expression, increase *Bax*/*Bcl-2* ratio, increase *Bax*/*Bcl-2* ratio, and induce caspase 3-mediated apoptosis by increasing the *Bax*/*Bcl-2* ratio and inducing caspase 3 activation.

When the results of our study were examined, it was found that the ratio of Bax and Bcl-2 in the cells to which the combination doses were applied changed in favour of apoptosis, compared to the cells in which both EA and etoposide were given alone. Therefore, EA may increase the apoptotic effects of etoposide and induce mitochondrial apoptosis by upregulating Bax and downregulating *Bcl-2* expression.

*Survivin* overexpression in malignant tumours is associated with a poor prognosis, shorter survival time, more aggressive behaviour, and higher resistance to treatment (Jaiswal et al. 2015; Chen 2017). To determine whether *survivin* expression can act as a marker in veterinary medicine, Uchide et al. (2005) reported the prognostic importance of survivin in their study on canine skin and subcutaneous tissue tumours. Similarly, Shoeneman et al. (2012) reported that survivin inhibition induced apoptosis in Abrams and D-17 canine OSA cell lines. Our study found a statistically significant reduction in the *survivin* gene in the D-17 canine OSA cell line in combination doses compared to cells treated with EA and ET alone (*P* < 0.001).

Lu et al. (2017) showed that the expression of NF-κβ protein in OSA was associated with the OSA apoptotic index and inhibited the apoptosis of OSA cells. Gong et al. (2017) reported that NF-κβ was highly expressed in OSA, which is important for the early diagnosis and prognosis of OSA. EA is a potent NF-κβ inhibitor and induces apoptosis through NF-κβ inhibition (Shakeri et al. 2018). In parallel with these findings, EA, in combination doses, increased the inhibition of the expression of *NF-κβ* in the current study.

Before experimental modelling and preclinical studies, *in vitro* studies performed at the molecular level on cells grown from specific cell lines play a critical role in the development of cancer treatment strategies. In the current study, the potential synergistic and/or additive effects of the chemotherapeutic agent, ET, and natural polyphenol, EA, were evaluated in a canine OSA cell line.

ET and EA exhibit anticancer activity in D-17 canine OSA cells when administered as monotherapy. However, the combination of these agents exhibited a synergistic effect, inducing a stronger response at the apoptotic level. This finding suggests that EA may be a promising candidate as an adjuvant in chemotherapy.

However, given that *in* *vitro* models do not accurately reflect the tumour microenvironment, angiogenesis, and pharmacokinetic parameters, the clinical validity of the results obtained may be limited. Further molecular analyses and *in vivo* animal models are required to validate these findings.

In summary, the combination of ET and EA is a potentially efficacious approach for future treatment protocols because of its ability to elicit an effective anticancer response at reduced dosages.
